# Pharmacoeconomic evaluation of direct oral anticoagulants for cancer-associated thrombosis: a systematic review

**DOI:** 10.3389/fpubh.2025.1498692

**Published:** 2025-04-28

**Authors:** Xiaoyan You, Yang Liu, Xianying Wang, Jiali Qin, Xiaomei Wang

**Affiliations:** ^1^College of Pharmacy, Hebei Medical University, Shijiazhuang, China; ^2^Department of Pharmacy, Hebei Medical University Third Hospital, Shijiazhuang, China

**Keywords:** direct oral anticoagulants, cancer-associated thrombosis, venous thromboembolism, cost-effectiveness analysis, systematic review

## Abstract

**Objective:**

To synthesize pharmacoeconomic evidence of prevention and treatment of venous thromboembolism (VTE) in cancer patients with direct oral anticoagulants (DOACs) and evaluate the quality of the studies.

**Methods:**

PubMed, Embase, Scopus, the Cochrane Library, the Center for Reviews and Dissemination Database, the Health Technology Assessment Database, and the China National Knowledge Infrastructure Database were searched to collect economic evaluations. The search covered publications from their inception until June 13, 2024. Study selection was conducted independently by two researchers, with discrepancies resolved through discussion. The quality of the studies were assessed using the Consolidated Health Economic Evaluation Reporting Standards 2022 checklist, and the basic characteristics of the included studies were summarized descriptively.

**Results:**

A total of 15 studies were included, covering different income level countries: the United States, Spain, China, the Netherlands, Canada, and Brazil. Economic evaluation results for prevention strategies varied in different countries. The baseline VTE incidence and drug costs will determine whether DOACs are worthwhile. For the treatment of VTE in cancer patients, DOACs were found to be more cost-effective compared to low molecular weight heparins (LMWHs) and warfarin, though the incremental cost-effectiveness ratio varied significantly across countries. However, there is still a lack of pharmacoeconomic studies based on direct evidence on which DOAC to choose for VTE treatment in cancer patients.

**Conclusion:**

The cost-effectiveness of DOACs for VTE in cancer patients has been proven. Further research is needed to determine the best choice of DOAC. Thromboprophylaxis in all cancer patients is not recommended. It is still necessary for clinicians to evaluate the risk of VTE. Pharmacoeconomic study results are significantly influenced by the drug costs, patient preferences, and income levels of different countries and regions. Economic decisions should be made according to the specific national background.

## 1 Introduction

Venous thromboembolism (VTE) refers to the partial or complete obstruction of veins caused by the abnormal coagulation of blood. It is characterized by its rapid onset and insidious presentation. Once the condition progresses, it can directly impact the patient’s quality of life and long-term prognosis. Cancer associated thrombosis (CAT) refers to VTE that manifests in individuals diagnosed with cancer, typically presenting as deep vein thrombosis (DVT) in the upper or lower extremities, as well as pulmonary embolism (PE) (1). Any factors that contribute to blood stasis, hypercoagulability, and vascular wall injury can increase the risk of VTE. In cancer patients, interactions between tumor cells and their products with the host can lead to a hypercoagulable state. Additionally, surgery, chemotherapy, anti-angiogenic therapy, epidermal growth factor receptor tyrosine kinase inhibitors therapy, hormone therapy, tumor compression of blood vessels, peripheral venous catheter placement, and long-term bed rest are all risk factors for VTE in cancer patients (2, 3). Therefore, the prevalence, morbidity, and mortality associated with VTE are higher in cancer patients (4). Compared to non-cancer patients, cancer patients are at approximately 4–7 fold increased risk of developing VTE, accounting for 20–30% of all new VTE events in the community (5, 6). The GARFIELD-VTE study enrolled patients from 28 countries worldwide, who had active cancer and a history of cancer. And the results showed that VTE was the second leading cause of death after disease progression (7).

CAT not only leads to an increase in patient hospitalization rates and delays in cancer treatment, significantly impacting their quality of life and survival, but also imposes additional economic burdens on patients and healthcare systems. Patients with CAT incur approximately 40–50% higher healthcare costs than those without CAT (4, 8). Retrospective cohort studies based in the United States have shown that cancer patients with VTE incurred significantly higher overall all-cause hospitalization costs (mean US$21,299 versus US$7,459 per patient), outpatient costs (US$53,660 versus US$34,232 per patient), and total health care costs (US$74,959 versus US$41,691 per patient) compared to cancer patients without VTE. Moreover, all-cause total health care costs increased with the escalating risk of VTE occurrence (9, 10).

According to the guidelines issued by the American Society of Clinical Oncology (11), the European Society for Medical Oncology (12), and the American National Comprehensive Cancer Network (13, 14), the recommended pharmacological treatments or prevention include warfarin, unfractionated heparin (UFH), low molecular weight heparins (LMWHs), fondaparinux, and direct oral anticoagulants (DOACs). Additionally, aspirin prophylaxis is recommended for patients with multiple myeloma.

The current landscape encompasses many pharmacoeconomic studies related to the treatment or prevention of CAT, and the results show significant variability in different countries and regions. Due to the advantages of DOACs such as oral administration, no food interactions, fixed dosing, and no need for regular monitoring of the international normalized ratio, their use is increasing. To understand the cost-effectiveness of DOACs in the treatment and prevention of CAT, this study aims to review and summarize the current pharmacoeconomic research results. Our study will provide evidence-based support for conducting research, health policy making, and promoting rational drug use.

## 2 Materials and methods

We adopted the Preferred Reporting Items for Systematic Review and Meta-analysis (PRISMA) statement for this systematic review (15).

### 2.1 Literature search

PubMed, Embase, Scopus, the Cochrane Library, the Center for Reviews and Dissemination Database, the Health Technology Assessment Database, and the China National Knowledge Infrastructure Database were searched to collect economic evaluations. The search encompassed publications from the inception of the databases until June 13, 2024. Our search strategy focused on key terms that corresponding to the predefined main domains of cost-effectiveness analysis, cancer, thrombosis, direct oral anticoagulant. The main search domains were linked using the Boolean operator ‘AND’, and the keywords of the same domain were connected using the Boolean operator ‘OR’. The detailed search strategy and results are shown in Supplementary Table S1.

### 2.2 Inclusion and exclusion criteria

After the literature search, a two-stage screening process was conducted. First, two researchers independently screened the titles and abstracts of all retrieved studies to exclude obviously irrelevant ones. Then, the full texts of the remaining studies were reviewed to determine their final eligibility based on the predefined inclusion and exclusion criteria.

Inclusion Criteria: (1) Types of studies: Economic evaluations of using DOACs for the prevention or treatment of VTE in cancer patients, including cost-effectiveness analysis, cost-utility analysis, cost–benefit analysis, and cost-minimization analysis. (2) Study Population: Cancer patients with or without VTE. (3) Intervention: Use of DOACs including dabigatran, rivaroxaban, apixaban, or edoxaban. (4) Comparator: Pharmacological therapies and prophylactic measures recommended in guidelines.

Exclusion Criteria: (1) Non-English literature. (2) Exclusion of reviews, theoretical or methodological introductions, and literature on research progress. (3) Exclusion of unpublished or incomplete information such as conference abstracts and letters. (4) Exclusion of duplicate literature. (5) Exclusion of literature that only includes cost analysis and budget impact analysis results.

### 2.3 Data extraction

Two researchers independently extracted literature data and cross-checked their findings. In event of disagreement, they conducted discussions or consultations to reach a consensus. The extracted content includes study characteristics regarding publication (author, year of publication, country), study design (target population, time horizon, comparators, model type, discount rate, outcome measure, perspective, cost type), and study results (cost, outcome, incremental cost-effectiveness ratio (ICER), subgroup analysis, willingness-to-pay threshold (WTP), sensitivity analysis).

### 2.4 Quality assessment

The quality of the included studies was evaluated by the Consolidated Health Economic Evaluation Reporting Standards 2022 (CHEERS 2022) checklist (16). It consists of 7 main categories with 28 items: (1) Title, (2) Abstract, (3) Introduction, (4) Methods, (5) Results, (6) Discussion, and (7) Other relevant information. The scoring criteria are as follows: for each item, 1 point was scored for full compliance, 0.5 point for partial compliance, 0 point for not compliance, and items not applicable were not counted. The quality assessment of the literature was independently evaluated by two researchers. In case of discrepancies, they were resolved through discussion or by seeking arbitration from a third researcher.

### 2.5 Data synthesis

Due to the differences in healthcare resource consumption across different countries and different perspectives of economic evaluations, there are substantial differences in the characteristics of the study subjects, model assumptions, measurements of costs and outcome parameters, as well as the design of economic models. Therefore, quantitative integration of data is not feasible. Instead, data extraction was analyzed and summarized through descriptions and tables.

## 3 Results

### 3.1 Literature search

**Figure 1:**
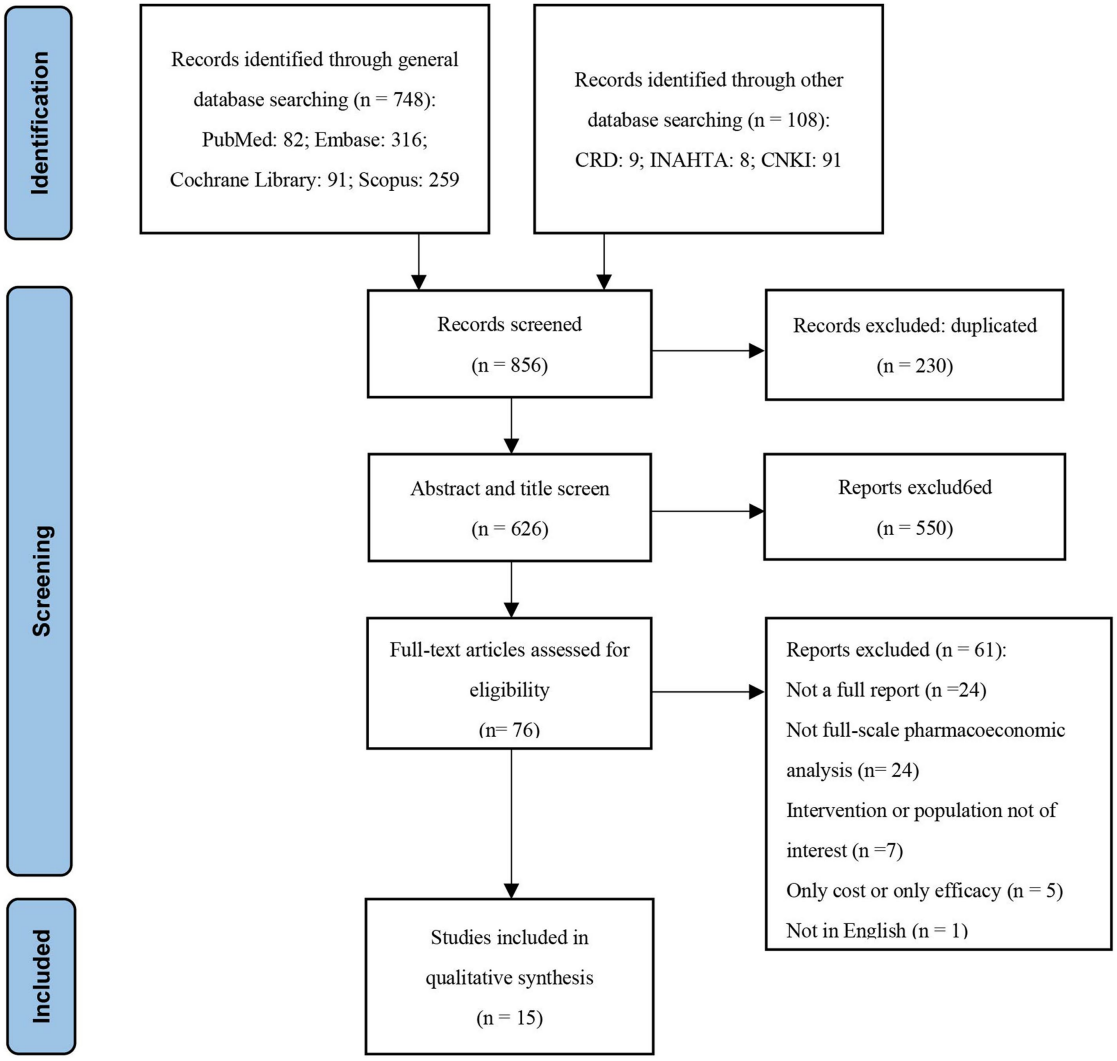
Flow chart and results of literature screening.

A total of 856 studies were identified using the search strategy and 230 duplicates were removed, leaving 626 studies for initial screening. Following further screening of titles and abstracts according to inclusion and exclusion criteria, 550 studies were excluded for not meeting the criteria. Subsequently, 76 studies underwent full-text review, of which 61 studies were excluded for the following reasons: (1) Not a full report (*n* = 24); (2) Not a comprehensive pharmacoeconomic analysis (*n* = 24); (3) Intervention or population not of interest (*n* = 7); (4) Only cost or only efficacy (*n* = 5); (5) Not in English (*n* = 1). This review included a total of 15 studies, and the study selection process is presented in a PRISMA flow chart (Figure 1).

### 3.2 General characteristics

The basic characteristics are presented in the Table 1. The studies included in this review were published between 2019 and 2023. Most of the studies were conducted in high-income countries, including the United States (17–24), Spain (25, 26), the Netherlands (27), and Canada (28). Only three studies were conducted in middle-income countries: Brazil (29) and China (30, 31). Among the included studies, eight focused on treatment and secondary prevention of VTE in cancer patients (17, 18, 22, 24, 25, 27, 29, 31), comparing the cost-effectiveness of DOACs (including apixaban, rivaroxaban, and edoxaban) with LMWHs, with one study also comparing with warfarin (29). The remaining focused on primary prevention in cancer patients at risk of VTE (19–21, 23, 26, 28, 30), mainly comparing the cost-effectiveness of DOAC prophylaxis with no thromboprophylaxis and LMWHs prophylaxis. All studies were cost-effectiveness analyses, with the time horizons ranging from 28 days to lifelong. Studies with time horizons exceeding 1 year applied annual discount rates ranging from 1.5 to 5% (18, 20–22, 24, 26–31). All studies utilized decision analysis models for pharmacoeconomic evaluations, employing Markov models or decision tree models, or a combination of short-term decision analysis models and long-term Markov models. Typically included health states were on anticoagulant treatment, off anticoagulant treatment, PE, DVT, intracranial hemorrhage (ICH), non-ICH major bleeding (MB), clinically relevant non-major bleeding, VTE-related death, MB-related death, and death by any cause, etc. Except for one study that did not report the perspective (19), all other studies reported the perspective of analysis, primarily from the public or private healthcare system perspective (20, 21, 24–26, 28–31), followed by the payer perspective (18, 23) and societal perspective (17, 27). One study concurrently used societal and healthcare system perspectives (22). Quality-adjusted life years (QALYs) were used as the outcome measure in all studies, while the ICER was used for comparing the values between treatment strategies.

**Table 1:** Basic features of the included studies.

Author/Publication year/Country	Target population	Time horizon	Comparators	Model type	Discount	Outcome	Perspective	Cost type	CHEERS score (%)
Connell, 2019; USA	Patients with general cancer or gastrointestinal malignancy who had acute symptomatic or incidental VTE	6 months	Edoxaban vs. dalteparin	Markov simulation model	NA	QALY	The US societal perspective	Direct costs and indirect costs	74.1%
Li, 2019; USA	65-year-old patients with active malignancy and first acute symptomatic CAT	60 months	DOAC vs. dalteparin	Markov state-transition model	3%	QALY and LY	Payer’s perspective	Direct medical costs	83.9%
de Jong, 2020; Netherlands	Cancer patients at risk of recurrent VTE	5 years	Rivaroxaban vs. dalteparin	Markov model	4%	QALY	Societal perspective	Direct costs and indirect costs	88.9%
Du, 2020;China	Cancer patients undergoing chemotherapy with Khorana score≥2	5 years	DOAC vs. no prophylaxis	Decision-tree and Markov model	3%	QALY	Chinese healthcare system perspective	Direct medical costs	82.1%
Glickman,2020; USA	People with gynecologic cancer after surgical resection	28 days	Apixaban vs. enoxaparin	Decision tree model	NA	QALY	NR	Direct medical costs	75.9%
Li, 2020; USA	Patients with cancer who are at intermediate-to-high risk for VTE	40 years	Low-dose DOAC vs. placebo	Markov state-transition model	3%	QALY and LY	Health sector perspective	Direct medical costs	83.9%
Lopes, 2020; Brazil	Active cancer population of 64 years-old, 70 kg and with an index VTE event	5 years	Edoxaban vs. LMWH; Edoxaban vs. warfarin	Markov state-transition model	5%	QALY	Brazilian private health system perspective	Direct medical costs	75.0%
Kimpton, 2021; Canada	Ambulatory patients with cancer who were starting chemotherapy and were at intermediate-to-high risk of VTE	Lifetime (20.6 years)	Apixaban primary thromboprophylaxis vs. usual care	Decision tree and a Markov model	1.5%	QALY and LY	Canada’s publicly funded health care system perspective	Direct medical costs	80.4%
Ryan, 2021; USA	Women initiating neoadjuvant chemotherapy for advanced-stage ovarian cancer with a Khorana VTE risk score ≥2.	36 months	Prophylactic DOAC taken for 18 weeks during chemotherapy vs. no VTE prophylaxis.	Decision tree model	3%	QALY	Health system perspective	Direct medical costs	78.6%
Wumaier, 2021; China	Cancer population of 64-year-old,70 kg, and with VTE event	6 months and 5 years	DOAC vs. LMWH	Markov state-transition model	5%	QALY	Chinese healthcare system perspective	Direct medical costs	83.9%
Muñoz, 2022; Spain	Active cancer outpatient suffering VTE	12 months	DOAC (apixaban, rivaroxaban, edoxaban) vs. LMWH	Markov state-transition model	NA	QALY and LY	Spanish healthcare system perspective	Direct medical costs	75.9%
Shin, 2022; USA	65-year-old patients with active malignancy and index VTE event	6 months and 60 months	DOAC vs. LMWH	Markov state-transition model	3%	QALY	The US healthcare system perspective and societal perspective	The US healthcare system perspective: direct costs; societal perspective: direct costs and indirect costs	83.9%
Bell, 2023; USA	Patients with newly diagnosed endometrial cancer who underwent minimally invasive staging surgery.	28 days	No extended pharmacologic vs. prophylactic enoxaparin vs. prophylactic apixaban	Decision tree model	NA	QALY	The US healthcare payor perspective	Direct medical costs	72.2%
Gulati, 2023; USA	Adult patients with cancer at the time they develop thrombosis	Lifetime	Apixaban vs. edoxaban vs. rivaroxaban vs. enoxaparin	Markov cohort state transition model	3%	QALY	Health care sector perspective	Direct medical costs	83.9%
Muñoz, 2023; Spain	Ambulatory cancer patients starting chemotherapy with an intermediate-to-high risk of VTE, Khorana score ≥2 points.	180 days and 5 years	Apixaban and rivaroxaban vs. no prophylaxis	A short-term decision analysis and subsequent long-term Markov model	3%	QALY	Spain’s National Health Service perspective	Direct medical costs	80.4%

QALY, quality-adjusted life year; LY, life-years; NR, not reported.

### 3.3 Results of cost-effectiveness analyses

Du et al. (30), Li et al. (20), Kimpton et al. (28), and Muñoz et al. (26) compared DOAC prophylaxis versus no thromboprophylaxis among cancer patients receiving chemotherapy with intermediate-to-high risk of VTE. These studies included apixaban 2.5 mg twice daily, rivaroxaban 10 mg daily, and edoxaban 60 mg daily as doses for DOAC prophylaxis. All studies utilized data from the AVERT (32) and CASSIN (33) trials, which showed that DOACs significantly reduce the incidence of VTE in cancer patients but may increase the risk of bleeding. The included studies demonstrated that DOACs result in higher QALYs compared to no prophylaxis. However, The results based in Canada (28) and Spain (26) found apixaban prophylaxis was more cost-saving compared to no prophylaxis, contrary to China (30) and the United States (20). When the WTP threshold was set at one time the per-capita gross domestic product (GDP) of China in 2019, apixaban prophylaxis was not cost-effective (30). However, the cost-effectiveness of DOACs for prophylaxis improved in cancer patients with a higher risk of VTE (Khorana score ≥3). In other studies, low-dose DOAC prophylaxis showed cost-effectiveness in patients with a modified Khorana score of 2 or more. Muñoz et al. (26) separately compared the cost-effectiveness of apixaban and rivaroxaban versus no prophylaxis. The result showed that compared to no prophylaxis, apixaban was dominant and rivaroxaban was cost-effective (with additional costs).

Ryan et al. (21) focused on patients with ovarian cancer receiving neoadjuvant chemotherapy, showing that DOAC prophylaxis was effective but more expensive than no prophylaxis, with an ICER exceeding the WTP. They also conducted an exploratory analysis on thromboprophylaxis using low-dose aspirin, which was found to be both more effective and less expensive than no prophylaxis, costing US$531 less per patient and resulting in slightly improved QALYs (0.000246) compared to no prophylaxis.

Glickman et al. (19) evaluated thromboprophylaxis in gynecologic cancer patients, and Bell et al. (23) assessed endometrial cancer patients undergoing minimally invasive hysterectomy. Both studies compared apixaban prophylaxis and enoxaparin prophylaxis, revealing that apixaban prophylaxis was not only less expensive but also more effective. Bell et al. (23) also compared thromboprophylaxis with no prophylaxis, finding that no prophylaxis was superior. Only when the risk of DVT is 14% or higher, apixaban prophylaxis for 28 days would be cost-effective compared with no prophylaxis. When the risk of DVT is 4.8% or higher, a 7-day course of apixaban prophylaxis was found to be cost-effective.

Eight studies focused on the treatment and secondary prevention of VTE in cancer patients who had already experienced VTE (17, 18, 22, 24, 25, 27, 29, 31). All studies compared the cost-effectiveness of DOACs and LMWHs, consistently demonstrating that DOACs were more cost-effective and represented cost-saving strategies. Among them, Lopes et al. (29) also compared the cost-effectiveness of DOACs with warfarin, showing that although warfarin was a more cost-saving strategy, the ICER of DOACs remained below WTP. Three studies (17, 18, 31) conducted subgroup analyses among patients with gastrointestinal malignancies, revealing that although DOACs increased the risk of MB, the lower overall costs resulted in their ICER remaining below the WTP compared to LMWHs.

Two studies compared apixaban, edoxaban, and rivaroxaban in different contexts. Muñoz et al. (25) showed that apixaban had lower costs and better clinical outcomes from a Spanish healthcare perspective. In contrast, Gulati et al. (24) analyzed Veterans Affairs Federal Supply Schedule pricing in the United States and found that although apixaban had lower costs, rivaroxaban was slightly more effective. When incorporating GoodRx data, which reflect commercial pharmacy expenditures, rivaroxaban demonstrated a cost-effectiveness advantage over apixaban.

### 3.4 Sensitivity analysis

All included studies conducted sensitivity analyses, with one-way deterministic sensitivity analyses (DSA) in 13 articles (18–25, 27–31), two-way DSA in 4 articles (17, 21, 24, 30), probabilistic sensitivity analyses (PSA) in 13 articles (18, 20–31), and scenario analyses in 8 articles (18, 20–22, 24, 27, 28, 30).

In the context of VTE prevention among cancer patients, the ICER of DOAC prophylaxis versus no prophylaxis is highly sensitive to the risk of VTE, but the results did not reverse (20, 28, 30). In China, the outcomes of DOACs versus no prophylaxis were reversed in populations with a Khorana score ≥3 (30). Muñoz et al. conducted a probability deterministic sensitivity analysis using the incremental net benefit instead of the ICER. The result showed that variations in the probability of cancer mortality with or without prophylaxis made DOACs no longer cost-effective (26). In PSA, there was a higher probability of cost-effectiveness for DOACs in the United States and Canada, at 94 and 99.87% respectively, but the probability is much lower in China and Spain, both <70% (20, 26, 28, 30).

Sensitivity analysis for VTE prevention among ovarian cancer patients receiving neoadjuvant chemotherapy (21) identified drug costs of DOACs, baseline risk of VTE, efficacy of DOACs, and VTE case mortality as factors potentially influencing the ICER of DOACs versus no prophylaxis.

Glickman et al. (19) and Bell et al. (23) showed that changes in all examined parameters did not reverse the results when comparing apixaban prophylaxis with no prophylaxis or enoxaparin prophylaxis under WTP. The results showed that no prophylaxis was favored in 41.1% of trials, apixaban prophylaxis was favored in 33.7% of trials, and enoxaparin prophylaxis was favored in 25.2% of trials (23).

In the comparison of DOACs and LMWHs for treating CAT patients, drug costs were the main driving factors in the United States background (17, 18, 22, 24). Li et al. (18) using a cheaper generic enoxaparin instead of dalteparin would result in the costs of LMWHs being close to those of DOACs, with the ICER below the WTP. Besides drug costs, the relative risk of cancer mortality, the relative utility values, and the probability of non-PE and non-MB death were influential parameters determining the ICER (18, 22). Gulati et al. calculated the costs and effects of apixaban, edoxaban, and rivaroxaban separately, with the sensitivity analysis showing that in real-world scenarios, apixaban would only be the most cost-effective treatment strategy if its monthly cost was below US$530 when the WTP was set at US$50,000 (24).

In other countries, drug costs did not significantly affect the economic outcomes. Parameter changes did not affect the result that DOACs were more cost-effective than LMWHs in Brazil and Spain (25, 29). In the Netherlands, changes in parameters such as the risk of MB, treatment duration, and the risk of VTE recurrence had the greatest impact on incremental costs, with the risks of MB and VTE recurrence significantly affecting incremental QALYs (27). From the perspective of the Chinese healthcare system, the ICER of DOACs and LMWHs was sensitive to the utility and the risk of MB occurrence, which could potentially reverse the economic study results (31).

In all PSA (18, 22, 24, 27), although the ICER might change with the aforementioned parameters, DOACs still had a higher probability of being more cost-effective compared to LMWHs. Lopes et al. (29) also conducted a sensitivity analysis comparing the cost-effectiveness of edoxaban and warfarin, finding that in all simulations, the incremental cost of edoxaban was below the WTP, proving the robustness of the study results.

The results of the included studies and the sensitivity analyses are presented in Table 2.

**Table 2:** Economic evaluation result of drug use of VTE prevention of cancer patients.

Author/Publication year/Country	Cost	Outcome	ICER	Subgroup analysis	WTP	Sensitivity analysis
Connell, 2019; USA	Edoxaban: $6,061 Dalteparin: $19,398	Edoxaban: 0.34 QALYs; Dalteparin: 0.35 QALYs	$1,873,535/QALY	Gastrointestinal malignancy: $694,058/QALY	$150,000/QALY	Two-way DSA: key drivers: the drug costs
Li, 2019; USA	Incremental costs: -$24,129	Incremental effectiveness: −0.04 QALYs; −0.04LYs	$623,459/QALY	Patients without gastrointestinal malignancies: $730,183/QALY	NR	One-way DSA: key drivers: the cost of dalteparin, the RR of cancer mortality, the utility values PSA; Scenario analysis
de Jong, 2020; Netherlands	Incremental costs: -€1,476	Incremental effectiveness: 0.012 QALYs	Negative	NR	NR	One-way DSA: key drivers: the risk of MB, treatment duration of dalteparin, recurrent VTE risks during the first 6 months PSA; Scenario analysis
Du, 2020;China	Incremental costs: -$930	Incremental effectiveness: 0.072	$12,919/QALY	Khorana = 0: $14,104/QALY Khorana = 1 or 2: $12,040/QALY Khorana≥3: $8,280/QALY	$10,276/QALY	One-way DSA: key drivers: the RR of death, the RR of symptomatic and asymptomatic VTE Two-way DSA; PSA; Scenario analysis
Glickman, 2020; USA	Incremental costs: -$27,014	Incremental effectiveness: 4.13 QALYs	Negative	NR	High value: < $50,000/QALY Low value: > $175,000/QALY	One-way DSA: no reasonable variation of parameters would have led to change the result
Li, 2020; USA	Incremental costs: $1,445	Incremental effectiveness: 0.16 LYs; 0.12 QALYs	$11,947/QALY	Khorana Score≥3: $5,794/QALY Khorana Score = 2: $15,118/QALY	$50,000/QALY	One-way DSA: key drivers: the RR of VTE, the RR of MB, drug costs PSA; Scenario analysis
Lopes, 2020; Brazil	Edoxaban vs. LMWHs: $16,654.27 Edoxaban vs. warfarin: $736.90	Edoxaban vs. LMWHs: 3.2 QALYs Edoxaban vs. warfarin: 0.29 QALYs	Model 1: $5,204.46/QALY Model 2: $2,541.03/QALY	NR	$22,738.21/ QALY	One-way DSA: no reasonable variation of parameters would have led to change the result PSA
Kimpton, 2021; Canada	Incremental costs: -$6972.84 CAD	Incremental effectiveness: 0.083 QALYs; 0.080 LYs	Negative	NR	Can$50,000/QALY	One-way DSA: no reasonable variation of parameters would have led to change the result PSA; Scenario analysis
Ryan, 2021; USA	Incremental costs: $1,620	Incremental effectiveness: 0.0063 QALYs	$256,218/QALY	NR	$100,000-150,000/QALY	One-way DSA: key drivers: DOACs drug costs; baseline VTE probability; DOACs effectiveness; VTE case mortality rate Two-way DSA; PSA; Scenario analysis
Wumaier, 2021; China	month: incremental costs: -$1064.66 year: incremental costs: -$1927.48	month: DOACs: incremental effectiveness:0.03 QALYs year: Incremental effectiveness: −0.02 QALYs	6-month: -$32,922.16/QALY 5-year: $112,895.50/QALY	Gastrointestinal malignancy: $32,821.83/QALY	$30,427.74/QALY	One-way DSA: key drivers: the utility values, non-ICH MB events PSA
Muñoz, 2022; Spain	DOAC: €1,994 (apixaban: €1,944; rivaroxaban: €2,122; edoxaban: €1,968) LMWHs: €2,512	DOACs: 0.54 QALYs; 0.77 LYs (apixaban: 0.55 QALYs; 0.79 LYs; rivaroxaban: 0.53 QALYs; 0.76 LYs; edoxaban 0.52 QALYs; 0.74 LYs) LMWHs: 0.53 QALYs;0.76 LYs	Apixaban dominant.	NR	€30,000/QALY	One-way DSA: no reasonable variation of parameters would have led to change the result PSA
Shin, 2022; USA	Incremental cost: -$9134.66 to -$15,281.92	Incremental effectiveness: 0.43–1.25 QALYs	Negative	NR	$50,000/QALY	One-way DSA: key drivers: the LMWHs utility value, the probability of non-PE and non-MB death, drug costs PSA; Scenario analysis
Bell, 2023; USA	No prophylaxis: $236.61 Apixaban: $328.71 Enoxaparin: $382.81	No prophylaxis: 0.062 QALYs; Apixaban: 0.058 QALYs; Enoxaparin: 0.050 QALYs	Negative	NR	$100,000/QALY	One-way DSA: no reasonable variation of parameters would have led to change the result PSA
Gulati, 2023; USA	Base-case (apixaban: $20,246; enoxaparin: $26,569; edoxaban: $28,207; rivaroxaban: $29,845) Real-world (apixaban: $31,868; enoxaparin: $32,334; edoxaban: $36,598; rivaroxaban: $36,674)	Base-case (apixaban: 2.3171 QALYs; enoxaparin: 2.2301 QALYs; edoxaban: 2.2405 QALYs; rivaroxaban: 2.3365 QALYs) Real-world (apixaban: 2.2405 QALYs; enoxaparin: 2.2301 QALYs; edoxaban: 2.3171 QALYs; rivaroxaban: 2.3365 QALYs)	API dominated. Rivaroxaban: $50,053/QALY to $493,246/QALY (compared to apixaban)	NR	$50,000/QALY	One-way DSA: monthly anticoagulant cost Two-way DSA; PSA; Scenario analysis
Muñoz, 2023; Spain	Apixaban: €1,077.10; No prophylaxis: €1,136.58 Rivaroxaban: €1,001.14; No prophylaxis: €884.91	Apixaban: 0.5380 QALYs; No prophylaxis: 0.5328 QALYs. Rivaroxaban: 0.5400 QALYs; No prophylaxis: 0.5338 QALYs.	Apixaban dominant. Rivaroxaban: €18,746.77/QALY.	NR	€25,000/QALY	Probabilistic deterministic sensitivity analysis: key drivers: the probability of cancer mortality with or without prophylaxis PSA

QALY, quality-adjusted life year; LY, life-years; ICER, incremental cost-effectiveness ratio; WTP, willingness-to-pay threshold; NR, not reported; RR, relative risk; HR, hazard ratio; DSA, deterministic sensitivity analysis; PSA, probabilistic sensitivity analysis.

### 3.5 Quality of the identified studies

The percentage of compliance with the items ranged from 72.2 to 88.9%. Details of the quality assessment are presented in the Supplementary Table S2. In the Methods category, less than 50% of the studies complied with the items Health economic analysis plan, Characterizing heterogeneity, Characterizing distributional effects, and Approach to engagement with patients and others affected by the study. Two studies (22, 24) described the health economic analysis plan they used, both based on the recommendations from the Second Panel on Cost-Effectiveness in Health and Medicine (34). Five studies (17, 18, 20, 30, 31) described patient heterogeneity through subgroup analyses within the study, showing how the results varied in specific populations. None of the studies described distributional effects. One study (27) mentioned that it was not suitable to involve patients or the public in the design, conduct, reporting, or dissemination plans, while the remaining reports did not mention whether there was patients or others involvement. Additionally, most reports (17, 18, 20–22, 24, 28, 30, 31) partially met the Setting and Location item under the Methods category, only stating the countries in which the studies were based without mentioning the particular healthcare setting or any other relevant sectors. Under the Results category, apart from de Jong et al. (27), which did not apply to the item Effect of engagement with patients and others affected by the study, none of the other studies mentioned relevant content.

## 4 Discussion

VTE is prevalent, highly burdensome, and associated with a risk of worse outcomes for patients with cancer (35). Currently, the mainstream recommended anticoagulants for the prevention and treatment of CAT include warfarin, parenteral anticoagulants (UFH, LMWHs, or fondaparinux), and DOACs. DOACs are recommended in guidelines for treating CAT due to their advantages, such as oral administration, fixed dosing, no need for laboratory monitoring, reduced patient discomfort, and improved adherence. The commonly used DOACs in clinical practice include apixaban, rivaroxaban, edoxaban, and dabigatran. Because the mechanism of action of dabigatran differs from that of factor Xa inhibitors like apixaban, and evidence for treating CAT is insufficient, its efficacy requires further confirmation through research (12).

Many clinical trials have demonstrated the efficacy and safety of DOACs in treating and preventing VTE in cancer patients. A meta-analysis showed that DOACs reduce the overall risk of VTE compared to LMWHs but increase the risk of bleeding without a significant difference in survival rates (36). Another study revealed that in patients with intermediate-to-high risk cancer undergoing chemotherapy, DOACs significantly reduced the overall incidence of VTE compared to placebo (risk ratio: 0.53, 95% confidence interval: 0.36–0.78, *p* = 0.001), without significantly increasing the risk of MB during the intervention period. However, there was no difference in all-cause mortality between the two groups (37). While the efficacy and safety of DOACs in cancer patients have been confirmed, the high cost of treatment and the rapid and insidious onset of VTE impose a heavy economic burden on patients. Therefore, selecting cost-effective treatment options is crucial.

Multiple economic studies have proven the cost-effectiveness of DOAC prophylaxis for cancer patients at intermediate-to-high risk of VTE (20, 26, 28, 30). However, the results are inconsistent even in the same health system perspective in the United States. It may be attributed to several factors: The researchers did not consider rare long-term consequences of VTE, such as post-thrombotic syndrome and chronic thromboembolic pulmonary hypertension. Some studies did not account for the negative impact of clinically relevant non-MB on quality of life. Moreover, some studies considered pooled estimates from both prevention and treatment VTE studies, whereas others only used pooled estimates from prevention trials, which may have led to the reversal of the economic study results (21).

Ryan et al. (21) and Muñoz et al. (26) indicated that the incremental QALYs of DOAC prophylaxis compared to no prophylaxis were minimal. When the utility-based generic quality of life difference is less than 0.03 units, it cannot be considered different from one another (38). Ryan et al. also conducted an additional study on aspirin prophylaxis, which showed that aspirin prophylaxis was more effective and less expensive than no prophylaxis. However, the incremental QALYs were too small to determine clinical relevance, requiring further research for validation.

Du et al. (30) and Muñoz et al. (26) indicated that the probability of DOACs being cost-effective was low (<70%). However, sensitivity analyses and scenario analyses targeting cancer patients with Khorana scores ≥3 showed that higher baseline VTE risk increased the likelihood of DOAC prophylaxis being cost-effective. Additionally, Muñoz et al. (26) separately compared the cost-effectiveness of apixaban and rivaroxaban with no prophylaxis, based on the results of the AVERT (32) and CASSINI (33) trials, respectively. Currently, there are no head-to-head clinical trials comparing apixaban and rivaroxaban, and further research is needed to determine which DOAC is more cost-effective.

Aside from the study of Ryan et al. (21), which focused solely on ovarian cancer patients undergoing neoadjuvant chemotherapy, the other studies did not stratify cancer patients. Further subgroup analyses based on different cancer types are necessary. All scenario analyses included in the studies were based on risk assessments using the Khorana model. However, the accuracy of this model in identifying high-risk patients is still questioned. Further research is needed to confirm whether there are more suitable risk assessment models to improve VTE risk prediction capabilities (39).

In the studies on the treatment and secondary prevention of cancer patients with existing VTE, all results supported the use of DOACs over LMWHs (17, 18, 22, 24, 25, 27, 29, 31). However, the incremental effectiveness of DOACs compared to LMWHs varied among studies, potentially due to the preferences of patients for the route of administration. Future studies should consider the value of patient preferences for DOACs and LMWHs, as any minor changes in utility weights could significantly alter the QALY outcomes between interventions.

Muñoz et al. (25) and Gulati et al. (24) separately calculated the costs and effects of apixaban, edoxaban, and rivaroxaban. The result indicated that apixaban is dominant over LMWH and other DOACs within the Spanish healthcare system perspective. However, scenario analysis based on the United States Department of Veterans Affairs Federal Supply Schedule and actual pharmacy costs showed that either apixaban or rivaroxaban is more cost-effective. Currently, there are no head-to-head comparisons of DOACs for efficacy and safety in CAT patients. The transition probabilities used in these two studies were derived from the randomized controlled clinical trials and a network meta-analysis (40–44), which lack direct comparative evidence and could introduce heterogeneity and bias. Therefore, more studies are needed to confirm the efficacy, safety, and cost-effectiveness among different DOACs.

In the context of high drug prices in the United States, drug costs are a primary driver of economic evaluation outcomes (17, 18, 22, 24). Using cheaper enoxaparin (generic) instead of dalteparin in studies can make LMWHs costs comparable to DOACs, making LMWHs more cost-effective (18). However, the first generics for apixaban were approved by the United States Food and Drug Administration in 2019, but will likely not be available until the patent expires in 2026, while the patent for rivaroxaban expires in 2024. Therefore, further pharmacoeconomic evaluations of generic drugs are needed (45–47).

All included pharmacoeconomic analysis reports were of good quality according to the CHEERS 2022 checklist, likely due to the recent publication dates and increasing emphasis on standardized reporting and transparency by researchers. However, few studies described the items of Health economic analysis plan, Characterizing distributional effects, Approach to engagement with patients and others affected by the study and Effect of engagement with patients and others affected by the study, which are newly added items in CHEERS 2022 compared to CHEERS 2013, emphasizing transparency and health equity (16, 48). The quality assessment results indicate that further efforts are needed to improve the transparency, comparability, and standardization of pharmacoeconomic evaluation reports.

There are still some limitations of the study. (1) Although a method of independent evaluation by two individuals was employed, some subjectivity is inevitable, as different researchers may have different interpretations of the checklist items, leading to biased assessments of literature quality. (2) The study only included English literature with full-text availability, potentially leading to information or data omissions. (3) The study only included countries such as the United States, Spain, and China. Due to differences in policies, drug costs, and distribution of different ethnicities among countries, the research results may not be fully applicable to other countries. (4) Due to the scarcity of eligible publications, diversity of evaluated molecules, and significant heterogeneity among identified studies, the integration of evaluation estimates as a whole was not possible, which is a common challenge in economic reviews (49). (5) The CHEERS checklist serves as a tool to assess the adherence of literature reports to writing standards rather than to evaluate the quality and evidence of research. Therefore, using CHEERS only represents an assessment of the clarity and completeness of pharmacoeconomic analysis report content rather than a quantitative assessment of the quality of research.

## 5 Conclusion

This study conducted a systematic review and quality evaluation of pharmacoeconomic studies on the prevention and treatment of CAT with DOACs. The results indicate that DOACs are more cost-effective than LMWHs in prevention and treating general CAT patients. However, DOAC prophylaxis is not recommended for all cancer patients without VTE; clinical decisions on thromboprophylaxis should be based on individual VTE risk assessments by clinicians. Furthermore, the selection of a specific DOAC requires additional pharmacoeconomic studies based on direct evidence.

Pharmacoeconomic outcomes are influenced by various factors such as drug costs, patient preferences, and economic conditions across different countries and regions. Thus, the results of these studies are not universally applicable. It is crucial for countries to conduct relevant pharmacoeconomic evaluations to obtain more localized and specific evidence. This evidence will support clinical decision-making and health policy development, ultimately promoting rational drug use and better health outcomes.

## Data Availability

The original contributions presented in the study are included in the article/Supplementary material, further inquiries can be directed to the corresponding author.
