# Supplementary material for: Pharmacoeconomic evaluation of direct oral anticoagulants for cancer-associated thrombosis: a systematic review
Source: Front Public Health. 2025 Apr 28;13:1498692. doi: 10.3389/fpubh.2025.1498692 (PMC12066465; doi:10.3389/fpubh.2025.1498692)
Supplement: Supplementary file 1 [file Table_1.DOCX]

**Supplementary Table S1 _Search Strategy**

| PubMed | | |
| --- | --- | --- |
| Search number | Query | Results |
| #1 | (((((((Neoplasms[MeSH Terms]) OR (neoplas*[Title/Abstract])) OR (malign*[Title/Abstract])) OR (cancer*[Title/Abstract])) OR (carcinoma*[Title/Abstract])) OR (adenocarcinoma*[Title/Abstract])) OR (tumour*[Title/Abstract])) OR (tumor*[Title/Abstract]) | 5,297,725 |
| #2 | ((((((((thromboembolism[MeSH Terms]) OR (thrombosis[MeSH Terms])) OR (Pulmonary Embolism[MeSH Terms])) OR (thrombo*[Title/Abstract])) OR (VTE[Title/Abstract])) OR (DVT[Title/Abstract])) OR (CAT[Title/Abstract])) OR (PE[Title/Abstract])) OR (embol*[Title/Abstract]) | 805,603 |
| #3 | (((((((((((((((factor xa inhibitors[MeSH Terms])) OR (dabigatran[MeSH Terms])) OR (direct oral anticoagulant*[Title/Abstract])) OR (non-vitamin K antagonist oral anticoagulant*[Title/Abstract])) OR (novel oral anticoagulant*[Title/Abstract])) OR (new oral anticoagulant*[Title/Abstract])) OR (DOAC*[Title/Abstract])) OR (NOAC*[Title/Abstract])) OR (apixaban*[Title/Abstract])) OR (betrixaban*[Title/Abstract])) OR (edoxaban*[Title/Abstract])) OR (rivaroxaban*[Title/Abstract])) OR (dabigatran*[Title/Abstract])) OR (ximelagatran*[Title/Abstract])) OR (Xa inhibitor*[Title/Abstract]) | 25,404 |
| #4 | (((((((Cost-Benefit Analysis[MeSH Terms]) OR (Cost-Effectiveness Analysis[MeSH Terms])) OR (cost benefit[Title/Abstract])) OR (cost effectiveness[Title/Abstract])) OR (cost utility[Title/Abstract])) OR (cost minimization[Title/Abstract])) OR (cost*[Title/Abstract])) OR (economic evaluation[Title/Abstract]) | 872,926 |
| #5 | #1 AND #2 AND #3 AND #4 | 82 |

| Embase | | |
| --- | --- | --- |
| Search number | Query | Results |
| #1 | 'neoplasms'/exp OR 'neoplasms' OR 'neoplasms':ti,ab OR 'neoplas*':ti,ab OR 'malign*':ti,ab OR 'cancer*':ti,ab OR 'carcinoma*':ti,ab OR 'adenocarcinoma*':ti,ab OR 'tumour*':ti,ab OR 'tumor*':ti,ab | 7,451,603 |
| #2 | 'thromboembolism'/exp OR 'thrombosis'/exp OR 'lung embolism'/exp OR 'thrombo*':ti,ab OR 'vte':ti,ab OR 'cat':ti,ab OR 'dvt':ti,ab OR 'pe':ti,ab OR 'embol*':ti,ab | 1,312,358 |
| #3 | 'blood clotting factor 10a inhibitor'/exp OR 'dabigatran'/exp OR 'direct oral anticoagulant*':ti,ab OR 'doac*':ti,ab OR 'noac*':ti,ab OR 'non-vitamin k antagonist oral anticoagulant*':ti,ab OR 'novel oral anticoagulant*':ti,ab OR 'new oral anticoagulant*':ti,ab OR 'apixaban*':ti,ab OR 'betrixaban*':ti,ab OR 'edoxaban*':ti,ab OR 'rivaroxaban*':ti,ab OR 'dabigatran*':ti,ab OR 'ximelagatran*':ti,ab OR 'xa inhibitor*':ti,ab | 133,591 |
| #4 | 'cost benefit analysis'/exp OR 'cost effectiveness analysis'/exp OR 'cost utility analysis'/exp OR 'cost minimization analysis'/exp OR 'economic evaluation'/exp OR 'cost benefit':ti,ab OR 'cost effectiveness':ti,ab OR 'cost utility':ti,ab OR 'cost minimization':ti,ab OR 'economic evaluation*':ti,ab | 396,816 |
| #5 | #1 AND #2 AND #3 AND #4 | 316 |

| Cochrane Library | | |
| --- | --- | --- |
| Search number | Query | Results |
| #1 | MeSH descriptor: [Neoplasms] explode all trees | 125,041 |
| #2 | neoplas* OR malign* OR cancer* OR carcinoma* OR adenocarcinoma* OR tumour* OR tumor* | 293,958 |
| #3 | MeSH descriptor: [Thromboembolism] explode all trees | 3,358 |
| #4 | MeSH descriptor: [Thrombosis] explode all trees | 7,016 |
| #5 | MeSH descriptor: [Pulmonary Embolism] explode all trees | 1,530 |
| #6 | thrombo* OR VTE OR CAT OR DVT OR PE OR embol* | 89,202 |
| #7 | MeSH descriptor: [Factor Xa Inhibitors] explode all trees | 900 |
| #8 | MeSH descriptor: [Dabigatran] explode all trees | 484 |
| #9 | direct oral anticoagulant* OR DOAC* OR NOAC* OR non-vitamin K antagonist oral anticoagulant* OR novel oral anticoagulant* OR new oral anticoagulant* OR apixaban* OR betrixaban* OR edoxaban* OR rivaroxaban* OR dabigatran* OR ximelagatran* OR Xa inhibitor* | 7,102 |
| #10 | MeSH descriptor: [Cost-Benefit Analysis] explode all trees | 11,443 |
| #11 | MeSH descriptor: [Cost-Effectiveness Analysis] explode all trees | 116 |
| #12 | MeSH descriptor: [Costs and Cost Analysis] explode all trees | 16,530 |
| #13 | cost benefit OR cost effectiveness OR cost utility OR cost minimization OR cost* OR economic evaluation* | 109,304 |
| #14 | (#1 OR #2) AND (#3 OR #4 OR #5 OR #6) AND (#7 OR #8 OR #9) AND (#10 OR #11 OR #12 OR #13)  in Cochrane Reviews | 91 |

| The international HTA database of the International Network of Agencies for Health Technology Assessment | | |
| --- | --- | --- |
| Search number | Query | Results |
| #1 | (Neoplasms)[mh] OR (neoplas*) OR (malign*) OR (cancer*) OR (carcinoma*) OR (adenocarcinoma*) OR (tumour*) OR (tumor*) | 4,155 |
| #2 | (Thromboembolism)[mh] OR (Thrombosis)[mh] OR (Pulmonary Embolism)[mh] OR (thrombo*) OR (VTE) OR (CAT) OR (DVT) OR (PE) OR (embol*) | 629 |
| #3 | (Factor Xa Inhibitors)[mh] OR (Dabigatran)[mh] OR (direct oral anticoagulant*) OR (DOAC*) OR (NOAC*) OR (non-vitamin K antagonist oral anticoagulant*) OR (novel oral anticoagulant*) OR (new oral anticoagulant*) OR (apixaban*) OR (betrixaban*) OR (edoxaban*) OR (rivaroxaban*) OR (dabigatran*) OR (ximelagatran*) OR (Xa inhibitor*) | 572 |
| #4 | #1 AND #2 AND #3 | 8 |

| [CRD Database](https://www.crd.york.ac.uk/CRDWeb/HomePage.asp) (include DARE, NHS Economic Evaluation Database, Health Technology Assessment Database) | | |
| --- | --- | --- |
| Search number | Query | Results |
| #1 | MeSH DESCRIPTOR Neoplasms EXPLODE ALL TREES IN DARE,NHSEED,HTA | 12,016 |
| #2 | (neoplas* OR malign* OR cancer* OR carcinoma* OR adenocarcinoma* OR tumour* OR tumor*) | 14,689 |
| #3 | MeSH DESCRIPTOR Thromboembolism EXPLODE ALL TREES IN DARE,NHSEED,HTA | 534 |
| #4 | MeSH DESCRIPTOR Thrombosis EXPLODE ALL TREES IN DARE,NHSEED,HTA | 671 |
| #5 | MeSH DESCRIPTOR Pulmonary Embolism EXPLODE ALL TREES IN DARE,NHSEED,HTA | 252 |
| #6 | (thrombo* OR VTE OR CAT OR DVT OR PE OR embol*) | 3,608 |
| #7 | MeSH DESCRIPTOR Factor Xa Inhibitors EXPLODE ALL TREES IN DARE,NHSEED,HTA | 77 |
| #8 | MeSH DESCRIPTOR Dabigatran EXPLODE ALL TREES IN DARE,NHSEED,HTA | 72 |
| #9 | (direct oral anticoagulant* OR DOAC* OR NOAC* OR non-vitamin K antagonist oral anticoagulant* OR novel oral anticoagulant* OR new oral anticoagulant* OR apixaban* OR betrixaban* OR edoxaban* OR rivaroxaban* OR dabigatran* OR ximelagatran* OR Xa inhibitor*) | 269 |
| #10 | #1 OR #2 | 15,550 |
| #11 | #3 OR #4 OR #5 OR #6 | 3,643 |
| #12 | #7 OR #8 OR #9 | 269 |
| #13 | #10 AND #11 AND #12 | 9 |

| Scopus | | |
| --- | --- | --- |
| Search number | Query | Results |
| #1 | ( TITLE-ABS-KEY ( neoplas* OR malign* OR cancer* OR carcinoma* OR adenocarcinoma* OR tumour* OR tumor* ) ) AND ( TITLE-ABS-KEY ( thrombo* OR vte* OR cat OR dvt OR pe OR embol* ) ) AND ( TITLE-ABS-KEY ( "direct oral anticoagulant*" OR doac* OR noac* OR "non-vitamin K antagonist oral anticoagulant*" OR "novel oral anticoagulant*" OR "new oral anticoagulant*" OR apixaban* OR betrixaban* OR edoxaban* OR rivaroxaban* OR dabigatran* OR ximelagatran* OR "Xa inhibitor*" ) ) AND ( TITLE-ABS-KEY ( "cost benefit" OR "cost effectiveness" OR "cost utility" OR "cost minimization" OR cost* OR "economic evaluation*" ) ) | 259 |

| CNKI | | |
| --- | --- | --- |
| Search number | Query | Results |
| #1 | (Title, abstract, keywords:"neoplasms" + "malignant" + "cancer" + "carcinoma" + "adenocarcinoma" + "tumour" + "tumor" ) AND (Title, abstract, keywords:"thromboembolism" + "thrombosis" +"VTE" + "CAT" + "DVT" + "PE" + "embolism") AND (Title, abstract, keywords:"oral anticoagulant" + "DOAC" + "NOAC" + "apixaban" + "betrixaban" + "rivaroxaban" + "dabigatran" + "Xa inhibitor") AND (Title, abstract, keywords:"cost benefit" + "cost effectiveness" + "cost utility" + "cost minimization" + "cost" + "economic evaluation") | 91 |

Date：2024-06-13

**Supplementary Table S2. Quality assessment of the included studies**

| CHEERS checklist item | | Connell 2019 | Li 2019 | de Jong 2020 | Du 2020 | Glickman 2020 | Li  2020 | Lopes 2020 | Kimpton 2021 | Ryan 2021 | Wumaier 2021 | Muñoz 2022 | Shin 2022 | Bell 2023 | Gulati 2023 | Muñoz 2023 |
| --- | --- | --- | --- | --- | --- | --- | --- | --- | --- | --- | --- | --- | --- | --- | --- | --- |
| 1 | Title | Y | Y | Y | Y | Y | Y | Y | Y | Y | Y | Y | Y | Y | Y | Y |
| 2 | Abstract | P | Y | Y | Y | Y | Y | Y | Y | Y | Y | Y | Y | Y | Y | Y |
| 3 | Background and objectives | P | Y | Y | Y | Y | Y | Y | Y | Y | Y | Y | Y | Y | Y | Y |
| 4 | Health economic analysis plan | N | N | N | N | N | N | N | N | N | N | N | Y | N | Y | N |
| 5 | Study population | Y | Y | Y | Y | Y | Y | Y | Y | Y | Y | Y | Y | Y | Y | Y |
| 6 | Setting and location | P | P | Y | P | Y | P | Y | P | P | P | Y | P | Y | P | Y |
| 7 | Comparators | Y | Y | Y | Y | Y | Y | Y | Y | Y | Y | Y | Y | Y | Y | Y |
| 8 | Perspective | P | Y | Y | P | N | Y | P | Y | P | Y | P | Y | P | Y | Y |
| 9 | Time horizon | P | Y | Y | Y | Y | Y | Y | Y | Y | Y | Y | Y | P | Y | P |
| 10 | Discount rate | NA | Y | Y | Y | NA | Y | Y | Y | Y | Y | NA | Y | NA | Y | Y |
| 11 | Selection of outcomes | Y | Y | Y | Y | Y | Y | Y | Y | Y | Y | Y | Y | Y | Y | Y |
| 12 | Measurement of outcomes | Y | Y | Y | Y | Y | Y | Y | Y | Y | Y | Y | Y | Y | Y | Y |
| 13 | Valuation of outcomes | Y | Y | Y | Y | Y | Y | Y | Y | Y | Y | Y | Y | Y | Y | Y |
| 14 | Measurement and valuation of resources and costs | Y | Y | Y | Y | Y | Y | Y | Y | Y | Y | Y | Y | Y | Y | Y |
| 15 | Currency, price date, and conversion | Y | Y | Y | Y | Y | Y | Y | Y | Y | Y | Y | Y | Y | Y | Y |
| 16 | Rationale and description of model | P | Y | Y | Y | Y | Y | Y | Y | Y | Y | Y | Y | Y | Y | Y |
| 17 | Analytics and assumptions | Y | Y | Y | Y | Y | Y | Y | Y | Y | Y | Y | Y | Y | Y | Y |
| 18 | Characterizing heterogeneity | Y | Y | N | Y | N | Y | N | N | N | Y | N | N | N | N | N |
| 19 | Characterizing distributional effects | N | N | N | N | N | N | N | N | N | N | N | N | N | N | N |
| 20 | Characterizing uncertainty | Y | Y | Y | Y | Y | Y | Y | Y | Y | Y | Y | Y | Y | Y | Y |
| 21 | Approach to engagement with patients and others affected by the study | N | N | Y | N | N | N | N | N | N | N | N | N | N | N | N |
| 22 | Study parameters | Y | Y | Y | Y | Y | Y | Y | Y | Y | Y | Y | Y | Y | Y | Y |
| 23 | Summary of main results | Y | Y | Y | Y | Y | Y | Y | Y | Y | Y | Y | Y | Y | Y | Y |
| 24 | Effect of uncertainty | Y | Y | Y | Y | Y | Y | Y | Y | Y | Y | Y | Y | Y | Y | Y |
| 25 | Effect of engagement with patients and others affected by the study | N | N | NA | N | N | N | N | N | N | N | N | N | N | N | N |
| 26 | Study findings, limitations, generalizability, and current knowledge | Y | Y | Y | Y | Y | Y | Y | Y | Y | Y | Y | Y | Y | Y | Y |
| 27 | Source of funding | Y | Y | Y | Y | P | Y | P | Y | Y | Y | Y | Y | P | Y | Y |
| 28 | Conflicts of interest | Y | Y | Y | Y | Y | Y | N | Y | Y | Y | N | Y | N | Y | Y |
| Total (%) | | 20/27 (74.1%) | 23.5/28 (83.9%) | 24/27 (88.9%) | 23/28 (82.1%) | 20.5/27 (75.9%) | 23.5/28 (83.9%) | 21/28 (75.0%) | 22.5/28 (80.4%) | 22/28 (78.6%) | 23.5/28 (83.9%) | 20.5/27 (75.9%) | 23.5/28 (83.9%) | 19.5/27 (72.2%) | 23.5/28 (83.9%) | 22.5/28 (80.4%) |

**Supplementary Table S3. PRISMA 2020 Main Checklist**

| **Topic** | **No.** | **Item** | **Location where item is reported** |
| --- | --- | --- | --- |
| **TITLE** | | | |
| **Title** | 1 | Identify the report as a systematic review. | Page 1 |
| **ABSTRACT** | | | |
| **Abstract** | 2 | See the PRISMA 2020 for Abstracts checklist | Page 1 |
| **INTRODUCTION** | | | |
| **Rationale** | 3 | Describe the rationale for the review in the context of existing knowledge. | Page 2 |
| **Objectives** | 4 | Provide an explicit statement of the objective(s) or question(s) the review addresses. | Page 2 |
| **METHODS** | | | |
| **Eligibility criteria** | 5 | Specify the inclusion and exclusion criteria for the review and how studies were grouped for the syntheses. | Page 3 |
| **Information sources** | 6 | Specify all databases, registers, websites, organisations, reference lists and other sources searched or consulted to identify studies. Specify the date when each source was last searched or consulted. | Page 2-3 |
| **Search strategy** | 7 | Present the full search strategies for all databases, registers and websites, including any filters and limits used. | Supplementary Table S1 |
| **Selection process** | 8 | Specify the methods used to decide whether a study met the inclusion criteria of the review, including how many reviewers screened each record and each report retrieved, whether they worked independently, and if applicable, details of automation tools used in the process. | Page 3 |
| **Data collection process** | 9 | Specify the methods used to collect data from reports, including how many reviewers collected data from each report, whether they worked independently, any processes for obtaining or confirming data from study investigators, and if applicable, details of automation tools used in the process. | Page 3 |
| **Data items** | 10a | List and define all outcomes for which data were sought. Specify whether all results that were compatible with each outcome domain in each study were sought (e.g. for all measures, time points, analyses), and if not, the methods used to decide which results to collect. | Page 3 |
|  | 10b | List and define all other variables for which data were sought (e.g. participant and intervention characteristics, funding sources). Describe any assumptions made about any missing or unclear information. | Page 3 |
| **Study risk of bias assessment** | 11 | Specify the methods used to assess risk of bias in the included studies, including details of the tool(s) used, how many reviewers assessed each study and whether they worked independently, and if applicable, details of automation tools used in the process. | Not applicable |
| **Effect measures** | 12 | Specify for each outcome the effect measure(s) (e.g. risk ratio, mean difference) used in the synthesis or presentation of results. | Page 3 |
| **Synthesis methods** | 13a | Describe the processes used to decide which studies were eligible for each synthesis (e.g. tabulating the study intervention characteristics and comparing against the planned groups for each synthesis (item 5)). | Page 3 |
|  | 13b | Describe any methods required to prepare the data for presentation or synthesis, such as handling of missing summary statistics, or data conversions. | Page 3 |
|  | 13c | Describe any methods used to tabulate or visually display results of individual studies and syntheses. | Page 3 |
|  | 13d | Describe any methods used to synthesize results and provide a rationale for the choice(s). If meta-analysis was performed, describe the model(s), method(s) to identify the presence and extent of statistical heterogeneity, and software package(s) used. | Page 3 |
|  | 13e | Describe any methods used to explore possible causes of heterogeneity among study results (e.g. subgroup analysis, meta-regression). | Page 3 |
|  | 13f | Describe any sensitivity analyses conducted to assess robustness of the synthesized results. | Page 3 |
| **Reporting bias assessment** | 14 | Describe any methods used to assess risk of bias due to missing results in a synthesis (arising from reporting biases). | Not applicable |
| **Certainty assessment** | 15 | Describe any methods used to assess certainty (or confidence) in the body of evidence for an outcome. | Page 3 |
| **RESULTS** | | | |
| **Study selection** | 16a | Describe the results of the search and selection process, from the number of records identified in the search to the number of studies included in the review, ideally using a flow diagram. | Figure 1 |
|  | 16b | Cite studies that might appear to meet the inclusion criteria, but which were excluded, and explain why they were excluded. | Page 4 |
| **Study characteristics** | 17 | Cite each included study and present its characteristics. | Table 1 and 2 |
| **Risk of bias in studies** | 18 | Present assessments of risk of bias for each included study. | Not applicable |
| **Results of individual studies** | 19 | For all outcomes, present, for each study: (a) summary statistics for each group (where appropriate) and (b) an effect estimate and its precision (e.g. confidence/credible interval), ideally using structured tables or plots. | Table 2 |
| **Results of syntheses** | 20a | For each synthesis, briefly summarise the characteristics and risk of bias among contributing studies. | Page 4-6 |
|  | 20b | Present results of all statistical syntheses conducted. If meta-analysis was done, present for each the summary estimate and its precision (e.g. confidence/credible interval) and measures of statistical heterogeneity. If comparing groups, describe the direction of the effect. | Page 4-6 |
|  | 20c | Present results of all investigations of possible causes of heterogeneity among study results. | Page 4-5 |
|  | 20d | Present results of all sensitivity analyses conducted to assess the robustness of the synthesized results. | Page 5 |
| **Reporting biases** | 21 | Present assessments of risk of bias due to missing results (arising from reporting biases) for each synthesis assessed. | Page 8-9 |
| **Certainty of evidence** | 22 | Present assessments of certainty (or confidence) in the body of evidence for each outcome assessed. | Page 6 |
| **DISCUSSION** | | | |
| **Discussion** | 23a | Provide a general interpretation of the results in the context of other evidence. | Page 7 |
|  | 23b | Discuss any limitations of the evidence included in the review. | Page 8 |
|  | 23c | Discuss any limitations of the review processes used. | Page 8-9 |
|  | 23d | Discuss implications of the results for practice, policy, and future research. | Page 7-8 |
| **OTHER INFORMATION** | | | |
| **Registration and protocol** | 24a | Provide registration information for the review, including register name and registration number, or state that the review was not registered. | The review was not registered |
|  | 24b | Indicate where the review protocol can be accessed, or state that a protocol was not prepared. | Page 2-3 |
|  | 24c | Describe and explain any amendments to information provided at registration or in the protocol. | Page 2-3 |
| **Support** | 25 | Describe sources of financial or non-financial support for the review, and the role of the funders or sponsors in the review. | Page 9 |
| **Competing interests** | 26 | Declare any competing interests of review authors. | Page 9 |
| **Availability of data, code and other materials** | 27 | Report which of the following are publicly available and where they can be found: template data collection forms; data extracted from included studies; data used for all analyses; analytic code; any other materials used in the review. | Page 13 |

*From:* Page MJ, McKenzie JE, Bossuyt PM, Boutron I, Hoffmann TC, Mulrow CD, et al. The PRISMA 2020 statement: an updated guideline for reporting systematic reviews. MetaArXiv. 2020, September 14. DOI: 10.31222/osf.io/v7gm2. For more information, visit: <www.prisma-statement.org>
